# Do oral combined contraceptive pills modify body image and sexual function?

**DOI:** 10.1186/s12958-022-00968-5

**Published:** 2022-06-28

**Authors:** Krzysztof Nowosielski

**Affiliations:** Department of Gynecology, Obstetrics and Gynecological Oncology, University Clinical Hospital, Medical University of Silesia, ul. Medykow 14, 40-752 Katowice, Poland

**Keywords:** Sexuality, Sexual dysfunction, Hormonal contraceptive, Sexual self-scheme, Body image

## Abstract

**Background:**

The effect of hormonal contraceptives on sexual function and body image is still controversial. Existing studies have not come to definite conclusions on the association between hormonal contraceptive use and sexual function/presence of sexual dysfunction or changes in body image perception. Thus, this study aimed to evaluate the prevalence of sexual problems/dysfunction in Polish women of reproductive age (18–45 years) and to assess to what extent oral combined contraceptive pills (OCCP) impact body image, sexual function and the prevalence of female sexual dysfunction (FSD).

**Methods:**

A total of 495 women were included in this cross-sectional questionnaire-based study. Sexual function was assessed by the Changes in Sexual Function Questionnaire (CSFQ), the prevalence of FSD was assessed by DSM-5 criteria, and body image was assessed by the Body Exposure during Sexual Activity Questionnaire (BESAQ). A total of 237 women using OCCP were the study group (HC), and the rest were controls (CG). A regression model was used to evaluate the influence of the selected variables on sexual function and the presence of FSD.

**Results:**

The prevalence of FSD was 7.5% in HC and 2.6% in CG, and 22% compared to 14% of women in HC and CG, respectively, reported sexual problems (CSFQ). The demographic characteristics of those using other contraception methods or not using any contraception (control group) were similar. The contraceptive group was characterized by significantly higher importance of sex (4.03 vs. 3.79), worse partner’s attitude toward sex (4.35 vs. 4.47), worse self-attitude toward sex (4.35 vs. 4.47), and worse body image (BESAQ) compared to controls. Among all of the variables, a lower level of anxiety (*t* = -1.99), positive attitudes toward sex (*t* = 2.05), watching erotic videos (*t* = 5.58) and a higher importance of sex (*t* = 5.66) were predictive of better sexual function (R2–0.38, F = 28.9, *p* = 0.0001).

**Conclusion:**

Sexual behaviors and function are different in those using OCCP compared to nonusers. The prevalence of sexual problems and dysfunction was higher in those using this hormonal method of contraception; however, using OCCP was not a risk factor for either worse sexual function or sexual dysfunction. Partners’ attitudes toward sex and general anxiety level were factors contributing to sexual function and the risk of sexual dysfunction in the population of women of reproductive age and should be routinely evaluated in clinical practice, especially before prescribing hormonal contraceptives.

## Background

According to the latest statement by the European Society for Sexual Medicine, “The effects of hormonal contraceptives on sexual function have not been well studied and remain controversial” [1]. The influence of contraceptives is definitely complex, as is women’s sexuality. There are numerous factors influencing the sexual response from testosterone levels in the brain and erogenic tissues through activation of β-adrenergic receptors in vaginal epithelial cells and the effectiveness of aquaporins [2–5] to the recently addressed androgen receptor polymorphism (CAG repeats length) [6–8]. However, none of these biological variables might have a dominant influence on sexual functioning, as other psychological, social and partner-related components play unquestionable roles (as shown in our other studies, e.g., in menopausal women) [9].

It is well established that serum androgen levels decline with age [10] and that hormonal contraceptives increase the level of sex hormone binding globulin (SHGB), which leads to a decrease in free testosterone (fT) levels [11]. However, a direct effect of that reduction of circulation androgens on sexual function has not been shown, either in observational studies [12] or interventional studies [13]. Additionally, a recent meta-analysis showed that better sexual function was associated with higher testosterone levels but not in those using hormonal contraceptives [14]. Similarly, no effect or a positive influence was noticed among women using long-lasting reversible contraceptive methods [15]. Neither any administration route [16] nor four-phasic agent [17] seemed to be superior.

As the results of available studies are conflicting, showing a negative (decreased libido, arousal and orgasm, increased pain) [18–20] or positive influence/no effect [1, 15, 21, 22], further studies are needed. Some new insight was brought by a paper by Wåhlin-Jacobsen et al. [8] showing that the number of CAG repeats in androgen receptors might play a role – longer repeats are associated with receptors more prone to changes in fT levels – and thus women with longer repeats could be more sensitive to changes in androgens due to the use of hormonal contraception. However, that dependency might not be linear but rather a “bell” shape. In that context, new well-designed studies with strict criteria for sexual dysfunction and functional assessments are needed to establish the real associations.

This study aimed to answer the following questions. First, what is the prevalence of sexual problems and sexual dysfunction based on DSM-5 criteria in the population of Polish women of reproductive age (18–45 years)? Second, to what extent do oral combined contraceptive pills (OCCP) influence sexual function (assessed by the Changes in Sexual Function Questionnaire—CSFQ) and the prevalence of Female Sexual Dysfunction—FSD (DSM-5 criteria). Third, what are the factors influencing the prevalence of FSD (DSM-5 criteria) and sexual function (assessed by CSFQ) in the general population of women of reproductive age and in those using OCCP? Fourth, how, in the subjective opinion of women, does OCCP influence sexual function? Finally, does dose, gestagen type, and regimen influence sexual function in women on hormonal contraception?

## Methods

A total of 1004 women of reproductive age (18–45 years) were eligible for this cross-sectional study conducted between March 1, 20,218 and December 31, 2020. The patients were recruited from among those attending routine gynecological yearly check-up visits and via the internet (Facebook). All were given a link to the online version of the questionnaire (based on www.surveymonkey.com) and were asked to fill it out at home. No identification data were collected. However, all participants had to reply “Yes” to a question agreeing to participate in this study.

The inclusion criteria were between 18 and 45 years old and agreeing to participate in the study. Women diagnosed with cancer or being treated for any cancer in the last 5 years, with severe cardiac insufficiency, less than 6 months after a myocardial infarction, unstable heart disease, uncontrolled arterial hypertension, in pregnancy or lactating, with a history of psychiatric disorders or currently on antidepressants, using progestin-only pills, with overactive bladder, prolapse of the vaginal vault more than grade 2, stress urinary incontinence, or who had never had vaginal/anal/oral sex were excluded from the study.

**Fig. 1:**
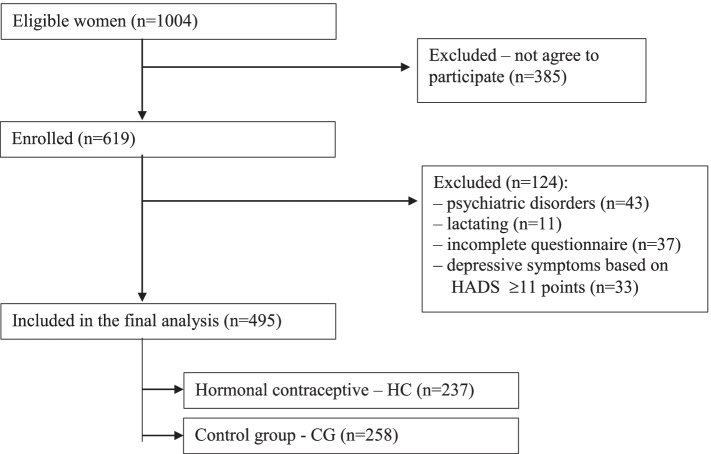
Study flow chart

From among all eligible women, 619 completed the questionnaire, and the response rate was 61.6%. Among those, 43 were being treated for a psychiatric disorder (depression), 11 were lactating, 37 only filled in the first page with demographic data, and 33 scored ≥ 11 points on the Hospital Depression and Anxiety Scale (HADS) scale, which was indicative of depressive symptoms. A total of 495 individuals were included in the final analysis (Fig. 1).

The study participants were divided into two groups: those using OCCP (HC group) and those using other contraceptive methods or not using any contraception (control group – CG).

The questionnaire used in the study contained general medical history questions, demographic and socioeconomic questions, and a battery of standardized and validated scales. Changes in the Sexual Function Questionnaire (CSFQ) were used to assess sexual function; scoring less than 41 points was indicative of sexual problems [9]. Based on the Diagnostic and Statistical Manual of Mental Disorders, 5th Edition (DSM-5), five questions were used to assess FSD [23]. The HADS was used to assess general anxiety levels and depressive symptoms. Body Exposure during Sexual Activity Questionnaire (BESAQ) was used for body image and sexual avoidance assessment [24]. Sexual satisfaction, attitudes toward sex, importance of sex, relationship quality, satisfaction from a partner as a lover, and sexual life quality were each assessed by a single 5-point Likert scale-based question. Finally, sexual self-schemas were evaluated by the Sexual Self-Schema Scale. Those who have negative schematics and a-schematics avoid emotional intimacy and have a lower level of desire and arousability. In contrast, those with positive schematics and co-schematics easily engage in sexual relationships and have a higher level of libido [25, 26]. A detailed description of all scales used may be found in our previous studies [9, 23–25]. Additionally, all women using contraception were asked if hormonal methods influenced their sexual function and the brand name and regimen of OCCP. Based on that information, the dose of estrogens and gestagen generation were identified. Additionally, the type and frequency of sexual activities were evaluated.

Risky sexual behaviors (RBS) were defined as “sexual contacts with more than one sexual partner at the same time, engaging in sexual activity with a casual person (one-night stand), frequent change of sexual partners, having intercourse with a person living with HIV, inconsistent use of condoms in oral, anal, and vaginal contacts except within the current relationship, prostitution or using the services of an escort agency, sexual contacts under the influence of psychoactive substances other than alcohol and marijuana, (chemsex) and drug injection with shared needles within the last 6 months” [9].

The study protocol was approved by the Ethical Committee of the Silesian Chamber of Physicians and Dentists in Katowice, Poland (decision number – ŚIL:/KB/755p/15).

Study sample calculation: according to statistical data collected in Poland in 2019, 8,943,200 women were of reproductive age. Based on this assumption, a minimum sample of 386 individuals was needed, with a 95% confidence level (CI) and a 5% margin of error. Additionally, assuming that the prevalence of FSD based on DSM-5 criteria in Poland would be 15% [23], the minimum required sample would be 454 with a CI of 95% and a margin of error of 3%. As at least 20% will be lost (not returning or not completing the questionnaires), the initial sample should be at least 544 women. Furthermore, a sample size of 425 was required to detect a difference of at least 0.3 between means on the CSFQ, with a value of 80% and a margin of error of 5%. Finally, post hoc analyses showed that the study had a power of 75% (α = 0.05) to detect a difference of 4.9% between groups in the prevalence of FSD (DSM-5).

Statistical analysis was performed with Statistica 12.0 for Windows (StatSoft, Warsaw, Poland). Missing values were assessed for all variables (less than 5%). Skewness and kurtosis were assessed to check for univariate and multivariate distribution normality. Values larger than 3 for skewness or larger than 10 for kurtosis were considered to indicate nonnormality [23]. To assess factors influencing sexual function (based on the CSFQ) and frequency of sexual dysfunction (DSM-5 criteria), univariate linear and logistic regression models were used. In the first step, all variables were checked for significant contributions to the assessed parameters. In the final step, only those statistically significant in the first step were introduced into the models developed using multivariate forward stepwise regression to establish the final models. P values less than 0.05 were defined as statistically significant.

## Results

The mean age of the studied population was 29.2 ± 7.2 (18.0–45.8) years old. A total of 237 (47.8%) women were using OCCP, 124 (25.0%) were using nonhormonal methods, and 135 (27.3%) were not using any contraception. In the nonhormonal group, 11.3% (*n* = 14) had nonhormonal IUDs, 24.1 (*n* = 30) were using natural planning methods, 24.1% (*n* = 30) were using condoms, and 42.7% (*n* = 52) were using withdrawal (not considered a contraceptive method in Europe but still in use in Poland).

The analysis of the type of OCCP showed that 50.3% (79%) were using contraceptives containing 4^th^ generation gestagens, 21.0% (*n* = 33) with 3^rd^ generation, 22.9% (36) with 1^st^ generation, and 5.7% (*n* = 9) with 2^nd^ generation. The most dominant proportion of women used 30 and 20 µg of estrogens (47.0% and 46.4%, respectively), with the 21 + 7 regimen being most prevalent (83.3%).

A higher proportion of women in the CG group declared excessive alcohol consumption, defined as more than one standard glass of wine, one beer or 50 ml of alcohol daily (61.5% vs. 38.5%); had never had oral sex (80.6% vs. 63.7%); were less sexually active in the last 6 months (91.5% vs. 97.9%); had less regular menses (75.6% vs. 88.6%); and had higher religiosity (2.66 vs. 2.44) compared to the HC group. The contraceptive group was characterized by a significantly higher number of pregnancies, higher importance of sex (4.03 vs. 3.79), worse partner’s attitude toward sex (4.35 vs. 4.47), and worse self-attitude toward sex (4.35 vs. 4.47) compared to the controls (Tables 1 and 2).

**Table 1:** General characteristics of the studied population – qualitative variables

Variable	Hormonal contraception	Controls	P^*^
Residency			
Urban	88.2 (209)	87.6 (226)	0.88
Rural	11.8 (28)	12.4 (32)	
Education			
Primary	0.4 (1)	1.56 (40)	0.18
Secondary	34.7 (82)	40.1 (103)	
Higher	64.8 (153)	58.4 (150)	
Education			
Black-collar	9.3 (22)	4.4 (37)	0.11
White-collar	62.4 (148)	58.9 (151)	
Unemployed	28.3 (67)	26.6 (68)	
Smoking (Yes)	18.6 (44)	17.8 (46)	0.82
Drugs (Yes)	10.1 (24)	9.3 (24)	0.75
Alcohol (Yes)	38.5 (62)	61.5 (99)	0.004
Religion			
Catholic	44.6 (138)	55.3 (171)	0.85
Other	39.5 (17)	60.5 (26)	
Atheist	57.4 (81)	42.5 (60)	
Participation in religious practices (yes)	45.3 (62)	54.7 (76)	0.47
Sexual initiation – oral sex (Yes)	63,7 (151)	80.6 (208)	0.0001
Sexual initiation – masturbation (Yes)	70.0 (166)	74.8 (193)	0.23
Being in RS (Yes)	81.4 (192)	78.7 (203)	0.44
Having sexual partner (Yes)	89.0 (211)	85.7 (221)	0.31
Sexual activity in last 6 months (Yes)	97.9 (232)	91.5 (236)	0.01
Watching erotic videos (Yes)	59.9 (142)	57.5 (111)	0.68
Sexual abuse in childhood (Yes)	5.5 (13)	5.2 (10)	0.89
Sexual behaviors			
WSW	0.4 (1)	2.4 (4)	0.14
WSWM	13.9 (33)	13.7 (23)	
WSM	85.7 (203)	83.9 (141)	
Sexual orientation			
Heterosexual	86.1 (204)	83.3 (140)	0.29
Homosexual	0.4 (1)	1.2 (2)	
Bisexual	13.5 (32)	14.3 (24)	
Asexual	0 (0)	1.2 (2)	
RSB (Yes)	49.4 (117)	36.8 (95)	
Regularity of menstruation (Yes)	88.6 (210)	75.6 (127)	0.001
Pregnancies (Yes)	32.1 (76)	32.7 (55)	0.88
Miscarriages (Yes)	1.3 (3)	1.9 (5)	0.81
Sexual distress – DSM-5 (Yes)	17.7 (40)	13.1 (20)	0.22
FSIAD – DSM-5 (Yes)	1.8 (4)	1.31 (2)	0.94
FOD – DSM-5 (Yes)	1.3 (3)	0.6 (1)	0.91
GPPPD – DSM-5 (Yes)	5.7 (13)	1.9 (3)	0.04
Lack of sexual satisfaction (Yes)	1.3 (3)	0.6 (1)	0.91
FSD – DSM-5 (Yes)	7.5 (17)	2.6 (4)	0.03
HADS—anxiety	9.4 (20)	14.5 (20)	0.10
Sexual problems—CSFQ	22.2 (26)	13.9 (26)	0.04
Pleasure—CSFQ	81.3 (152)	68.4 (80)	0.01
Desire/Frequency—CSFQ	19.8 (37)	35.9 (42)	0.01
Desire/Interest—CSFQ	33.6 (63)	41.0 (48)	0.24
Arousal/Excitement—CSFQ	89.8 (163)	79.5 (93)	0.02
Orgasm/Completion—CSFQ	62.6 (117)	45.3 (53)	0.01
Sexual self-Schema—positive	26.4 (43)	37.1 (36)	0.04
Sexual self-Schema – negative	36.2 (59)	17.5 (17)	0.001
Sexual self-Schema – A-schematic	14.7 (24)	10.3 (10)	0.2
Sexual self-Schema – Co-schematic	22.7 (37)	35.1 (34)	0.04

*RS* relationship, *RSB* Risky Sexual Behaviors, *WSW* woman who has sex with women, *WSM* woman who has sex with men, *WSWM* woman who has sex with women and men, *CSFQ* Changes in Sexual Function Questionnaire, *FSD* Female Sexual Dysfunction, *FISAD* Female Sexual Interest/Arousal Disorder, *FOD* Female Orgasmic Disorder, *GPPPD* Genito-Pelvic Pain/Penetration Disorder, *HADS* Hospital Anxiety and Depression Scale

^*^Chi squared test

**Table 2:** General characteristics of the studied population – quantitative variables

Variable	Controls	Hormonal contraception	P*
Age	28.79 (17.78–45.84) ± 7.35	29.59 (19.34–45.65) ± 6.95	0.10
Nr of cigarettes a day	7.50 (0.00–20.00) ± 6.58	6.82 (0.00–20.00) ± 4.18	0.59
BMI	22.50 (15.94–36.44) ± 3.43	22.59 (17.01–29.74) ± 2.58	0.14
Religiosity	2.66 (1.00–5.00) ± 1.18	2.44 (1.00–5.00) ± 1.14	0.04
Feeling physically attractive	3.29 (1.00–5.00) ± 0.99	3.40 (1.00–5.00) ± 0.92	0.23
Vaginal sex	1.04 (1.00–2.00) ± 0.20	1.00 (1.00–2.00) ± 0.07	0.46
Age of first genital sex	18.87 (14.00–33.00) ± 2.89	18.47 (13.00–24.00) ± 2.22	0.54
Age of first oral sex	18.91 (13.00–33.00) ± 3.08	18.74 (13.00–30.00) ± 2.58	0.78
Age of first masturbation	15.60 (8.00–30.00) ± 3.49	15.52 (8.00–30.00) ± 3.09	0.50
Importance of sex	3.79 (1.00–5.00) ± 0.91	4.03 (2.00–5.00) ± 0.73	0.01
Duration of RS	7.41 (0.50–26.00) ± 6.20	6.68 (0.50–25.00) ± 5.41	0.48
RS quality	4.53 (1.00–5.00) ± 0.79	4.45 (1.00–5.00) ± 0.73	0.07
Nr of lifetime male sexual partners	0.27 (0–6) ± 0.81	0.29 (0–10) ± 0.99	0.76
Nr of lifetime female sexual partners	4.42 (3.0–50.0) ± 6.18	4.7 (0.0–40.0) ± 6.19	0.81
Satisfaction from a partner as a lover	3.90 (1.00–5.00) ± 1.01	3.78 (1.00–5.00) ± 0.88	0.05
Partner's attitude toward sex	4.47 (1.00–5.00) ± 0.77	4.33 (1.00–5.00) ± 0.75	0.02
Attitudes toward sex	4.47 (3.00–5.00) ± 0.62	4.35 (2.00–5.00) ± 0.57	0.04
Vaginal sex/month	5.98 (0.00–30.00) ± 6.34	9.50 (0.00–30.00) ± 6.09	0.00
Cuddling/month	7.66 (0.00–50.00) ± 8.13	6.15 (0.00–40.00) ± 7.35	0.05
Anal sex/month	0.89 (0.00–14.00) ± 2.41	0.87 (0.00–20.00) ± 2.17	0.28
Oral sex/month	5.94 (0.00–40.00) ± 6.71	4.65 (0.00–81.00) ± 7.37	0.01
Mutual masturbation/month	4.06 (0.00–25.00) ± 5.48	3.56 (0.00–20.00) ± 4.12	0.38
Self-masturbation/month	3.42 (0.00–30.00) ± 5.15	4.27 (0.00–103.00) ± 8.00	0.01
Orgasm/month	10.46 (0.0–100.0) ± 11.36	8.57 (0.00–70.00) ± 8.98	0.11
nr of sexual events/month	14.69 (0.00–51.00) ± 38.18	10.80 (1.00–114.00) ± 11.39	0.20
Satisfying sex/months	8.92 (0.00–40.00) ± 7.29	9.05 (0.00–49.00) ± 8.22	0.78
Quality of sexual life	4.37 (1.00–5.00) ± 1.10	4.37 (1.00–5.00) ± 1.01	0.80
Day of cycle	18.75 (0.02–93.33) ± 15.71	16.66 (0.00–71.00) ± 10.81	0.81
Length of cycle	30.76 (20.00–90.00) ± 8.21	28.83 (20.00–90.00) ± 7.90	0.00
Length of bleeding	5.21 (3.00–10.00) ± 1.15	4.78 (1.00–56.00) ± 4.27	0.00
Nr of deliveries	0.33 (0.00–4.00) ± 0.73	0.60 (0.00–4.00) ± 1.00	0.02
Duration of contraception use	43.92 (3.00–300.0) ± 67.26	38.46 (3.00–276.0) ± 50.74	0.81
HADS—anxiety	7.69 (3.00–16.00) ± 2.84	6.98 (2.00–15.00) ± 2.50	0.05
HADS—depression	3.48 (0.00–10.00) ± 2.84	3.05 (0.00–10.00) ± 2.54	0.23
BESAQ	1.00 (0.07–3.36) ± 0.77	1.34 (0.00–3.57) ± 0.80	0.00
CSFQ—pleasure	3.90 (1.00–5.00) ± 1.09	3.81 (1.00–5.00) ± 0.83	0.10
CSFQ—desire/frequency	7.06 (2.00–10.00) ± 1.46	7.35 (4.00–10.00) ± 1.08	0.13
CSFQ—desire/interest	9.90 (5.00–15.00) ± 2.43	10.11 (3.00–14.00) ± 2.05	0.40
CSFQ—arousal/excitement	10.85 (5.00–15.00) ± 2.00	10.19 (3.00–15.00) ± 1.95	0.01
CSFQ—orgasm/completion	11.12 (3.00–15.00) ± 2.95	10.79 (3.00–15.00) ± 2.20	0.03
CSFQ—sum	46.09 (24.00–58.00) ± 6.33	46.35 (20.00–61.00) ± 5.51	0.82

*BESAQ* Body Exposure during Sexual Activity Questionnaire, *CSFQ* Changes in Sexual Function Questionnaire, *HADS* Hospital Anxiety and Depression Scale

^*^U Mann–Whitney test

Additionally, nonhormonal contraception users and those not using any contraception were similar except for a statically significant difference in the prevalence of excessive alcohol consumption (48.4% in the nonhormonal group vs. 28.9% in the no contraception group, *p* = 0.002), engaging in RBS (43.5 vs. 30.4%, respectively, *p* = 0.03), having a sexual partner (91.1% vs. 80.7%, respectively, *p* = 0.04), and frequency of vaginal sex a month (8.0 vs. 4.2, respectively, *p* = 0.001).

The analysis of sexual behaviors, functions, problems and dysfunction revealed that the HC group had a higher frequency of vaginal sex, lower frequency of oral sex, higher self-masturbation, better orgasm/completion but lower arousal/excitement function (based on the CSFQ), a lower proportion of sexual negative and co-schematic and a higher frequency of negative schematic women, and worse body image (higher sexual avoidance based on BESAQ) (Tables 1 and 2).

Sexual dysfunction was diagnosed (DSM-5) in 21 women (5.1%). That prevalence was higher in those using hormonal contraceptives than in controls (7.5% vs. 2.6%). However, the difference was only significant for cases of pain during sexual acts (5.7% vs. 1.9%). There was no difference in the presence of sexual distress.

**Fig. 2:**
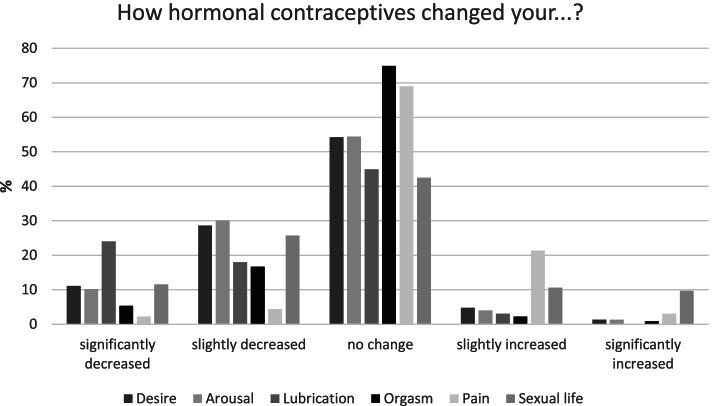
Subjective evaluation of hormonal contraceptives influence on sexual function

Sexual problems (based on the CSFQ) were noted in 17.1% (*n* = 52) of the studied women. The prevalence of such problems was higher in the HC group than in the control group. Low pleasure, low arousal/excitement and low orgasm/completion were more prevalent in the hormonal contraception group (Table 1). Additionally, when asked for subjective opinions, no changes or slight decreases (except for pain that slightly increased) were noted in women using hormonal methods (Fig. 2).

Finally, factors influencing sexual function and dysfunction were evaluated in a logistic model. The results showed that among all variables, a lower level of anxiety (*t* = -1.99), positive attitudes toward sex (*t* = 2.05), being schematic positive (*t* = -2.92), watching erotic videos (*t* = 5.58) and a higher importance of sex (*t* = 5.66) were predictive of better sexual function (corrected R2–0.38, F = 28.9, *p* = 0.0001). Using OCCP did not enter the model. In a similar analysis of only women using OCCP, similar factors were identified: positive attitudes toward sex (*t* = 2.43), being schematic positive (*t* = -3.12), watching erotic videos (*t* = 4.21) and higher importance of sex (*t* = 3.42) were predictive of better sexual function (corrected R2–0.35, F = 20.1, *p* = 0.0001). Neither gestagen generation, estrogen dose, regimen, nor duration of use influenced sexual function.

Further logistic regression showed two risk factors for sexual dysfunction (DSM-5) in the general population and three in the HC group, respectively: worse partner’s attitude toward sex (OR = 0.23; CI: 0.1–0.55; *p* = 0.01) and higher level of general anxiety (OR = 1.36; CI: 1.01–1.69; *p* = 0.01); and lower satisfaction from a partner as a lover (OR = 0.09; CI: 0.01–0.22; *p* = 0.02), worse partner’s attitude toward sex (OR = 0.16; CI: 0.03–0.94; *p* = 0.04), and higher level of general anxiety (OR = 1.61; CI: 1.06–2.43; *p* = 0.02). Using OCCP and none of the contraception related factors entered the model. Finally, the logistic regression model showed only one risk factor for GPPPD – low satisfaction from a sexual partner (OR = 0.19, CI: 0.04–0.94; p-0.04). Using OCCP did not enter the model.

## Discussion

To our knowledge, this is one of the first papers to use strict DSM-5 criteria to assess the prevalence of sexual dysfunction in the Polish population of women of reproductive age, especially those using OCCP. Additionally, partner-related factors, sexual self-schemas and body avoidance were also evaluated, providing a picture of the multidimensional dependencies in the regulation of sexual function in that group of women. The results might serve as a background for discussions on implementing interventions for anxiety, body image and attitudes toward sex when women inquire about using hormonal contraceptives.

Based on these study results, the prevalence of FSD in the general population was 5.1%, and in those using OCCP, it was 7.5%. These prevalences are much lower than that in our previous study of the general population (14.7%) [23]; however, women in the present report were younger (mean age 29 vs. 39, respectively), which might influence the results. A similar proportion of FSD based on the Female Sexual Function Index (FSFI) was found in a Finnish population [22]. No previous studies have evaluated FSD based on DSM-5 criteria.

A recent meta-analysis of studies on hormonal contraceptives and sexual dysfunction reported that based on FSFI scores, up to 36.7% of hormonal contraceptive users reported some sexual problems [18, 27]. This is in line with our results showing that 22% of users reported sexual problems. When analyzing the prevalence of disturbances in a particular domain, the numbers were much higher; however, that has to be interpreted with caution, as the scale might not be suitable for assessing sexual problems and the FSFI might be a better screening tool [21].

Some differences were also noted between users and nonusers in sexual behaviors – those in the HC group were more frequently sexually active, had more vaginal sex a month but had a lower frequency of orgasms. This is in line with other studies showing fewer orgasms (*t* =  − 2.39, *P* < 0.05), a higher rate of lubrication problems (*t* = 2.00, *P* < 0.05) and a lower frequency of pleasure (− 1.95, *P* < 0.05), with no differences in pain (in contrast to our study results) [20]. Despite the aforementioned differences between users and nonusers, the regression model showed no influence of hormonal contraceptives on either sexual function or the prevalence of dysfunction. This is similar to a recently published summary of conducted studies reporting increases or no change in desire, a decreased orgasm frequency (except among Mirena users), and no impact on vulvovaginal symptoms or lubrication [1, 21, 22]. No effect of estrogen dose or regimen, similar to our study, was noted in other papers [28]. In contrast, some papers showed an increased likelihood of worsening sexual desire (OR = 2.47), arousal (OR = 2.85) and sexual function in general (OR = 2.01) as assessed by the FSFI after 3 months of using drospirenone-containing oral contraceptives [21, 29]. However, the population included in that study was too small to draw a definite conclusion. Some differences might also be due to cultural factors and ethnicity – women from Europe seem to have a higher prevalence of pain during intercourse when using hormonal contraceptives compared to women on other continents [21].

When asked about their subjective opinions on the influence of hormonal contraceptives, women declared no effect or a slight decrease in sexual response, which is in line with some previous observations – no effect in 55% of users [30].

Interestingly, women using hormonal contraceptives had a worse body image (higher sexual avoidance based on the BESAQ), a higher frequency of pain during sex and a higher number of children than controls. However, no differences in the rates of sexual events with satisfaction and orgasm were noted. In contrast, the prevalence of sexual problems with pleasure, arousal and orgasm based on the CSFQ was higher in the OCCP group. This finding illustrates the complexity of sexual function. On the one hand, having more children who need to be taken care of and a higher frequency of pain could be a distractive factor – the frequency of orgasm and satisfactory sexual events would be expected to be lower. In this study, the numbers were equal between OCCP users and controls. On the other hand, the frequency of sexual problems was higher in hormonal contraceptive users. Those problems were, however, not distressing. This could also be explained by the motivation to use hormonal contraceptives. Those with a higher number of children and higher sexual avoidance, as described in previous studies [25], could be motivated to use contraceptives to protect against unplanned pregnancies or increased self-consciousness [31] despite possible pain during sex. The other possible explanation for the increase in pain was women using OCCP for the treatment of endometriosis, but that was not assessed in this study.

Body image was also assessed in the context of sexual function and contraceptive use; women in the contraceptive group had more avoidant sexual behaviors than controls. However, body perception during sexual activity was not a risk factor for sexual dysfunction or impaired sexual function. In previous studies, sexual function was worse in those expressing dissatisfaction with their bodies [32, 33]. It might be speculated that, as described in a previous study by Nowosielski et al., higher sexual avoidance may lead to contraceptive use. After eliminating the fear of unintended pregnancy, women may become more open to sexual cues and thus be more satisfied during sex [31]. However, some other factors were important in predicting sexual function and the risk of dysfunction, namely, anxiety level, watching erotic videos, the importance of sex, and the partners’ attitude toward sex. It seems, as presented in Rausch and Rettenberger’s recent paper, that partner-related factors might play a major role [34]. Similarly, the use of erotic material [34], the importance of sexual acts [35] in a positive way and anxiety in a negative way [36] were also reported to be influential by other authors.

Undoubtedly, hormonal contraceptives might influence some aspects of sexuality, such as the perception of partner attractiveness, thus modifying sexual receptivity or changing the concentration of oxytocin during the sexual cycle [1, 37]. However, the extent to which sexuality might be modified seems to be complex and depends on genetic and partner-related factors (having a partner, attitude toward sex) [35, 38]. In that context, an effort has to be made to educate women before introducing contraceptives or when sexual dysfunction emerges during hormonal contraceptive use. Motivations to use contraceptives, sexual self-schemes and body image perception should also be carefully discussed. It seems reasonable, as motivations for using hormonal contraceptives might not only lie in family planning but also might be rooted in psychological issues and sociocultural and sexual scripts that have to be modified before starting contraception. Changing the contraceptive preparation [13] or, if indicated, adding androgens [39] might be an option when sexual problems/dysfunctions emerge during contraceptive use. However, educational interventions and anxiety reduction could be better alternatives.

The paper also has some limitations. First, sexual satisfaction and relationship quality were assessed by a single question. However, a similar methodology was used in other studies and is generally accepted [40]. Second, the propensity for inhibition/excitation could also be evaluated, which is currently receiving increasing attention [23]. Third, we did not evaluate the effect of progestin-only pills (POP), hormonal IUDs, patches, rings or injections. In the Hassanin et al. study, 95% and 84% of injectable contraceptive and POP users reported a decline in sexual function [30]. Finally, a prospective study with a sexual function evaluation before and after using hormonal contraceptives would be more reliable in showing a causative association. Despite these limitations, the large number of participants and the validated instruments used in this study allows it to make valuable contributions to the current knowledge of hormonal contraceptives’ effect on sexuality.

## Conclusions

Sexual behaviors and function are different in those using hormonal contraceptives compared to nonusers. The prevalence of sexual problems and dysfunction is higher in those using hormonal methods; however, using hormonal contraceptives is not a risk factor for either worse sexual function or sexual dysfunction. Partners’ attitudes toward sex and the general anxiety level are factors contributing to sexual function and the risk of sexual dysfunction in the population of women of reproductive age and should be routinely evaluated in clinical practice, especially before prescribing hormonal contraceptives.

## Data Availability

The datasets used and/or analyzed during the current study are available from the corresponding author on reasonable request.

## Abbreviations

OCCP: Oral combined contraceptive pill
RS: Relationship
RSB: Risky sexual behaviors
WSW: Woman who has sex with women
WSM: Woman who has sex with men
WSWM: Woman who has sex with women and men
CSFQ: Changes in sexual function questionnaire
FSD: Female sexual dysfunction
FISAD: Female sexual interest/arousal disorder
FOD: Female orgasmic disorder
GPPPD: Genito-pelvic pain/penetration disorder
HADS: Hospital anxiety and depression scale
